# EP4 inhibition attenuates the development of diabetic and non-diabetic experimental kidney disease

**DOI:** 10.1038/s41598-017-03237-3

**Published:** 2017-06-13

**Authors:** Karina Thieme, Syamantak Majumder, Angela S. Brijmohan, Sri N. Batchu, Bridgit B. Bowskill, Tamadher A. Alghamdi, Suzanne L. Advani, M. Golam Kabir, Youan Liu, Andrew Advani

**Affiliations:** Keenan Research Centre for Biomedical Science and Li Ka Shing Knowledge Institute of St. Michael’s Hospital, Toronto, Ontario, Canada

## Abstract

The therapeutic targeting of prostanoid subtype receptors may slow the development of chronic kidney disease (CKD) through mechanisms that are distinct from those of upstream COX inhibition. Here, employing multiple experimental models of CKD, we studied the effects of inhibition of the EP4 receptor, one of four receptor subtypes for the prostanoid prostaglandin E_2_. In streptozotocin-diabetic endothelial nitric oxide synthase knockout mice, EP4 inhibition attenuated the development of albuminuria, whereas the COX inhibitor indomethacin did not. In Type 2 diabetic db/db mice, EP4 inhibition lowered albuminuria to a level comparable with that of the ACE inhibitor captopril. However, unlike captopril, EP4 inhibition had no effect on blood pressure or hyperfiltration although it did attenuate mesangial matrix accumulation. Indicating a glucose-independent mechanism of action, EP4 inhibition also attenuated proteinuria development and glomerular scarring in non-diabetic rats subjected to surgical renal mass ablation. Finally, *in vitro*, EP4 inhibition prevented transforming growth factor-ß1 induced dedifferentiation of glomerular podocytes. In rodent models of diabetic and non-diabetic CKD, EP4 inhibition attenuated renal injury through mechanisms that were distinct from either broadspectrum COX inhibition or “standard of care” renin angiotensin system blockade. EP4 inhibition may represent a viable repurposing opportunity for the treatment of CKD.

## Introduction

Prostanoids have long been appreciated as playing complex roles in renal (patho)physiology, functioning in the regulation of salt and water balance, renal blood flow, glomerular hemodynamics and renin release^1^. More recently, these oxidized metabolites of arachidonic acid that are generated by the cyclo-oxygenase (COX) enzyme system have also begun to be appreciated as being important regulators of glomerular filtration barrier permselectivity^2–4^. COX inhibition itself, for instance, reduces proteinuria in patients with kidney disease^5^. However, by diminishing renal blood flow and intraglomerular pressure it may also precipitate acute kidney injury in predisposed individuals^6^. Whether therapeutically targeting pathway members that lie downstream of the COX enzymes themselves can alter the natural history of kidney disease remains uncertain.

The five major prostanoids (prostaglandin E_2_ [PGE_2_], PGI_2_ (prostacyclin), PGD_2_, PGF_2α_ and thromboxane A_2_ [TXA_2_]) exert their effects through specific G-protein-coupled receptors, that themselves can exist as multiple different subtypes. For instance, four different receptor subtypes respond to PGE_2_ and they are designated EP1, EP2, EP3 and EP4^7^. The most widely expressed EP receptor is EP4^8^ and the most widely produced prostanoid in the body is PGE_2_^9^. Several reports employing genetic knockout or overexpression studies have revealed that EP receptor subtypes have different effects in different renal cells. By way of example, knockout of EP4 from vascular smooth muscle cells exacerbated renal injury^10^; EP4 overexpression in mesangial cells accelerated matrix production^11^; disruption of EP4 in the collecting duct impaired urinary concentration^12^; and mice lacking EP4 from their podocytes exhibited diminished glomerular scarring after renal mass ablation^13^.

Given the disparate cell-type dependent actions of EP4, it has been unclear what the global consequences of pharmacological EP4 (ant)agonism may be in kidney disease, with effects appearing to be at least in part dependent upon the model and agents employed. Pharmacological *agonism* of EP4, for example, attenuated folic acid-induced renal injury^14^, but exacerbated diabetes-associated kidney injury in mice^15^. In light of the apparently beneficial effects of EP4 deletion in glomerular cells^11, 13^ and in light of the pressing need for new treatments, here we hypothesized that EP4 *antagonism* would attenuate chronic kidney disease (CKD) development, including kidney disease due to diabetes the most common cause of kidney failure^16^. To test this hypothesis, we examined the effect of the EP4 antagonist ONO-AE3-208 (4-{4-Cyano-2-[2-(4-fluoronaphthalen-1-il) propionylamino] phenyl} butyric acid; *K*_i_ values EP4 1.2 nM, EP3 30 nM, FP 790 nM, TP 2,400 nM and >10,000 nM for other prostanoid receptors^17^) in three different rodent models and we compared its effects to broadspectrum COX inhibition and “standard of care” renin angiotensin system (RAS) blockade.

## Results

### The EP4 inhibitor ONO-AE3-208 attenuates albuminuria in streptozotocin-diabetic eNOS knockout mice

**Figure 1:**
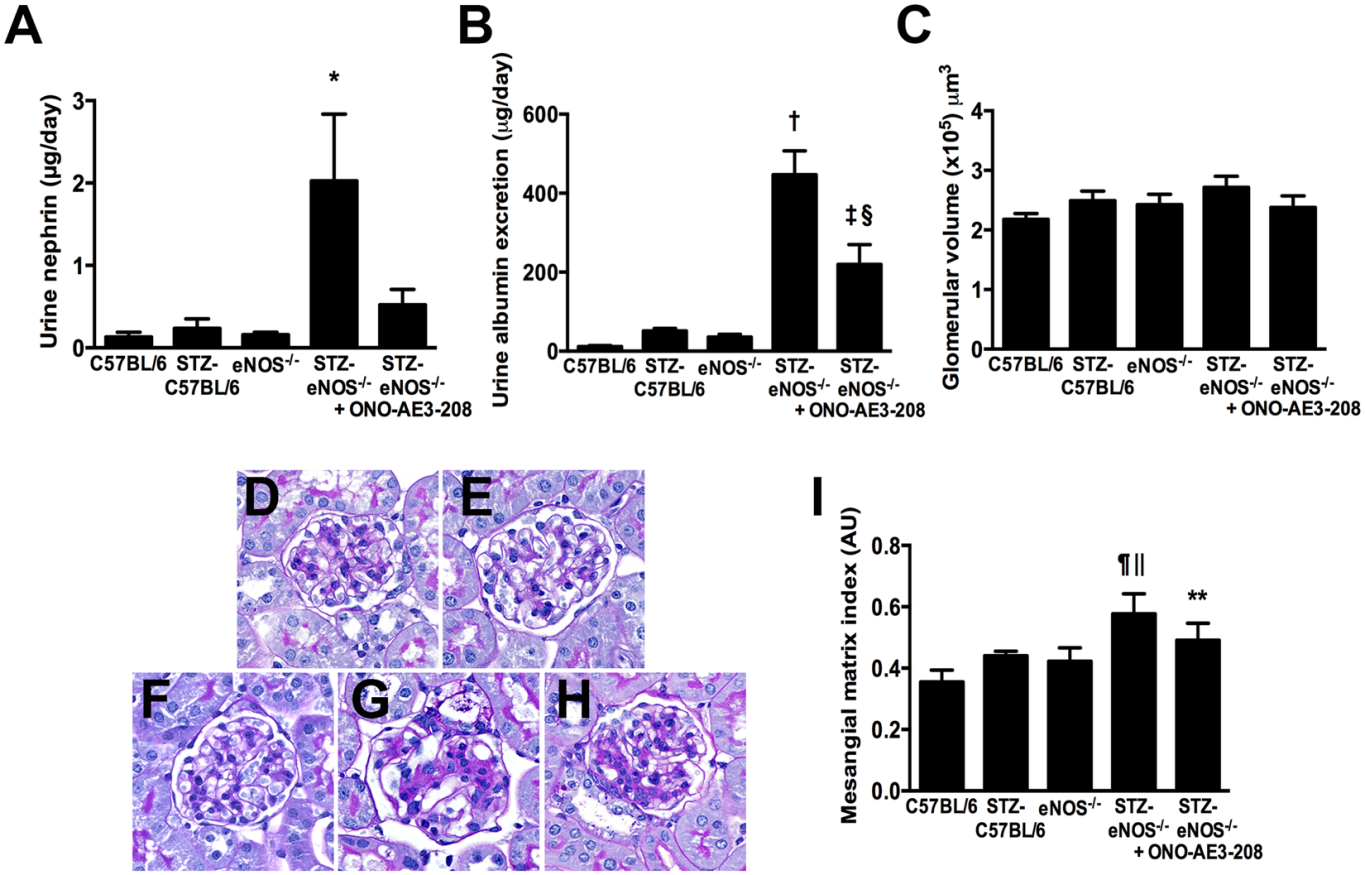
Effect of ONO-AE3-208 in non-diabetic and streptozotocin (STZ)-diabetic eNOS knockout (eNOS^−/−^, C57BL/6 genetic background) mice (n = 6/group). (**A**) Urine nephrin excretion after two weeks. (**B**) Urine albumin excretion after three weeks. (**C**) Glomerular volume after three weeks. (**D**–**H**) Representative photomicrographs of periodic acid-Schiff stained kidney sections from (**D**) C57BL/6 + vehicle, (**E**) STZ-C57BL/6 + vehicle, (**F**) eNOS^−/−^ + vehicle, (**G**) STZ-eNOS^−/−^ + vehicle, (**H**) STZ-eNOS^−/−^ + ONO-AE3-208. Original magnification ×400. (I) Mesangial matrix index. AU = arbitrary units. *p < 0.01 vs. all other groups; ^†^p < 0.0001 vs. C57BL/6 + vehicle, STZ-C57BL/6 + vehicle, or eNOS^−/−^ + vehicle; ^‡^p < 0.001 vs. C57BL/6 + vehicle or STZ-eNOS^−/−^ + vehicle; ^§^p < 0.01 vs. STZ-C57BL/6 + vehicle or eNOS^−/−^ + vehicle; ^¶^p < 0.01 vs. C57BL/6 + vehicle; ^||^p < 0.05 vs. STZ-C57BL/6 + vehicle or eNOS^−/−^ + vehicle; **p < 0.05 vs. C57BL/6 + vehicle;.

To determine whether inhibition of EP4 affects the development of experimental diabetic kidney disease, we initially performed studies in streptozotocin (STZ)-diabetic eNOS knockout (eNOS^−/−^) mice that develop massive albuminuria very soon after the induction of diabetes^18^. Wildtype (C57BL/6) and eNOS^−/−^ mice were made diabetic with STZ and were treated with ONO-AE3-208 (10 mg/kg/day) in drinking water beginning with the first intraperitoneal (i.p.) injection of STZ and continued for three weeks (Table 1). In comparison to their non-diabetic counterparts, body weight was lower in STZ-diabetic wildtype and eNOS^−/−^ mice (Table 1). After three weeks of diabetes, kidney weight was increased in STZ-eNOS^−/−^ mice but not in STZ-C57BL/6 mice, whereas kidney weight and kidney weight:body weight ratio were significantly lower in STZ-eNOS^−/−^ mice treated with ONO-AE3-208 than in vehicle-treated STZ-eNOS^−/−^ mice (Table 1). Two weeks after the first i.p. injection of STZ, urinary nephrin content was increased >10-fold in vehicle-treated STZ-eNOS^−/−^ mice, whereas it was approximately 50% lower in ONO-AE3-208-treated mice (Fig. 1A). By three weeks, urinary albumin excretion rate (AER) had increased >40-fold in STZ-eNOS^−/−^ mice compared to non-diabetic C57BL/6 mice, whereas AER was approximately 50% lower in ONO-AE3-208 treated STZ-eNOS^−/−^ mice than vehicle-treated STZ-eNOS^−/−^ mice (Fig. 1B). As expected, at this early stage of diabetes, glomerular volume was marginally albeit non-significantly increased in STZ-diabetic mice in comparison to their non-diabetic counterparts (Fig. 1C). Mesangial matrix index was also marginally increased in STZ-eNOS^−/−^ mice (Fig. 1D–I) and, although both glomerular volume and mesangial matrix index were numerically lower with ONO-AE3-208 treatment, changes in neither of these parameters achieved statistical significance.

**Table 1:** Metabolic characteristics of non-diabetic and streptozotocin (STZ)-diabetic wildtype (C57BL/6) and eNOS^−/−^ mice (C57BL/6 genetic background) and STZ-eNOS^−/−^ mice treated with ONO-AE3-208 for three weeks.

	*n*	Body weight (g)	Mean kidney weight (g)	Mean kidney weight: body weight (%)	Blood glucose (mmol/L)
C57BL/6	6	24.8 ± 0.4	0.152 ± 0.005	0.61 ± 0.01	9.9 ± 0.3
STZ-C57BL/6	6	22.3 ± 0.2^a^	0.159 ± 0.006	0.71 ± 0.03^e^	23.5 ± 0.9^a^
eNOS^−/−^	6	24.2 ± 0.5^b^	0.130 ± 0.006^ef^	0.54 ± 0.02^cj^	9.9 ± 0.7^c^
STZ-eNOS^−/−^	6	19.7 ± 0.3^acd^	0.156 ± 0.003^g^	0.81 ± 0.02^abd^	29.3 ± 1.7^adf^
STZ-eNOS^−/−^ + ONO-AE3-208	6	19.8 ± 0.4^acd^	0.140 ± 0.006^hi^	0.71 ± 0.03^edk^	31.6 ± 1.0^acd^

^a^p < 0.0001 vs. C57BL/6, ^b^p < 0.01 vs. STZ-C57BL/6, ^c^p < 0.0001 vs. STZ-C57BL/6, ^d^p < 0.0001 vs. eNOS^−/−^, ^e^p < 0.01 vs. C57BL/6, ^f^p < 0.001 vs. STZ-C57BL/6, ^g^p < 0.01 vs. eNOS^−/−^, ^h^p < 0.05 vs. STZ-C57BL6, ^i^p < 0.05 vs. STZ-eNOS^−/−^, ^j^p < 0.05 vs. C57BL^/^6^, k^p < 0.01 vs. STZ-eNOS^−/−^.

**Figure 2:**
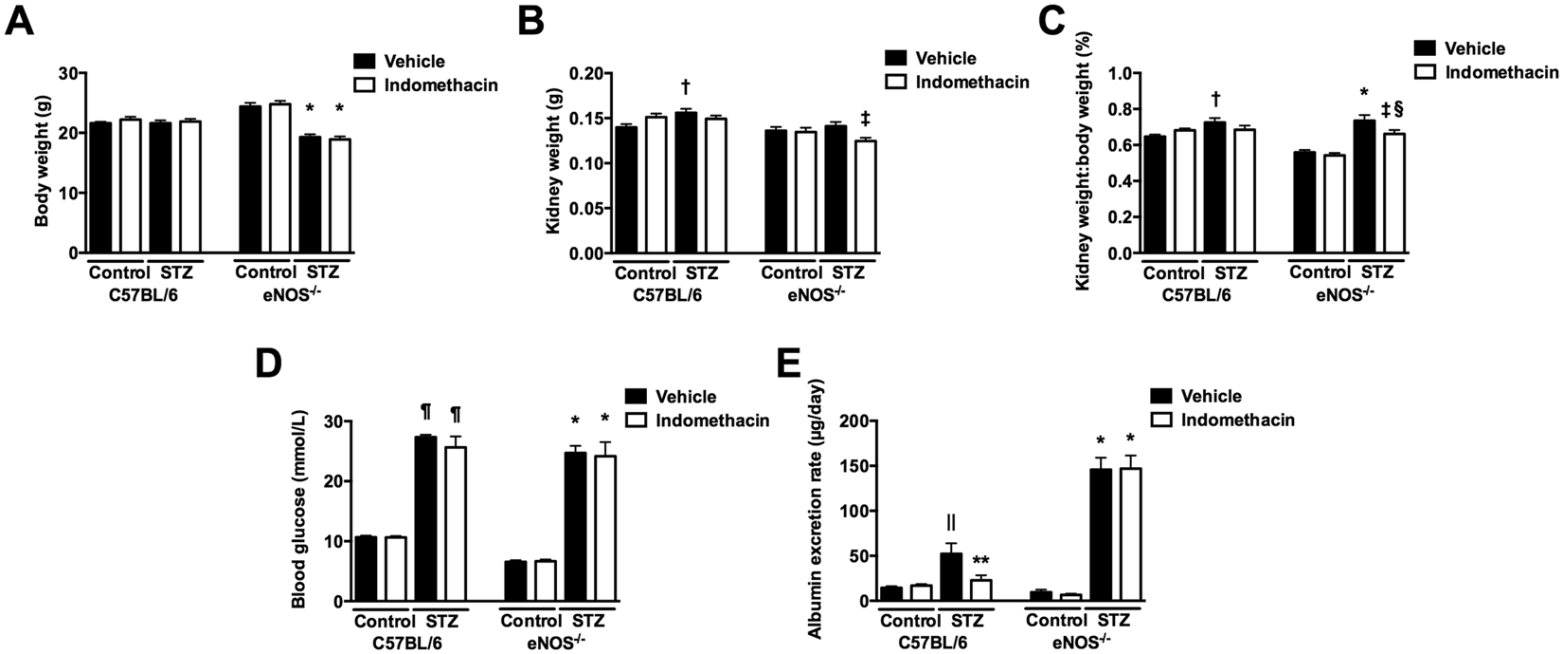
Effect of treatment with indomethacin for two weeks in non-diabetic and streptozotocin (STZ)-diabetic wildtype (C57BL/6) and eNOS knockout (eNOS^−/−^) mice (n = 10/group). (**A**) Body weight. (**B**) Kidney weight. (**C**) Kidney weight:body weight ratio. (**D**) Blood glucose. (**E**) Urine albumin excretion. *p < 0.0001 vs. eNOS^−/−^ + vehicle or eNOS^−/−^ + indomethacin; ^†^p < 0.01 vs. C57BL/6 + vehicle; ^‡^p < 0.05 vs. STZ-eNOS^−/−^ + vehicle; ^§^p < 0.01 vs. eNOS^−/−^ + vehicle or eNOS^−/−^ + indomethacin; ^¶^p < 0.0001 vs. C57BL/6 + vehicle or C57BL/6 + indomethacin; ^||^p < 0.001 vs. C57BL/6 + vehicle or C57BL/6 + indomethacin; **p < 0.01 vs. STZ-C57BL/6 + vehicle.

Because COX inhibition may also be anti-proteinuric^5^, but is ultimately associated with a generally adverse renal profile^6^, we compared the effect of ONO-AE3-208 in STZ-eNOS^−/−^ mice to that of the broadspectrum COX inhibitor, indomethacin (Fig. 2). Interestingly, indomethacin reduced albuminuria in STZ-C57BL/6 mice but not in STZ-eNOS^−/−^ mice (Fig. 2E). In contrast, even at the two week timepoint, albuminuria was already lower in ONO-AE3-208 treated STZ-eNOS^−/−^ mice than vehicle treated STZ-eNOS^−/−^ mice (AER [µg/day], vehicle 176 ± 34, ONO-AE3-208 97 ± 24, p < 0.05), the collective results indicating that the actions of EP4 inhibition are qualitatively different to those of broadspectrum COX inhibition.

### ONO-AE3-208 reduces albuminuria and mesangial matrix accumulation in db/db mice

**Figure 3:**
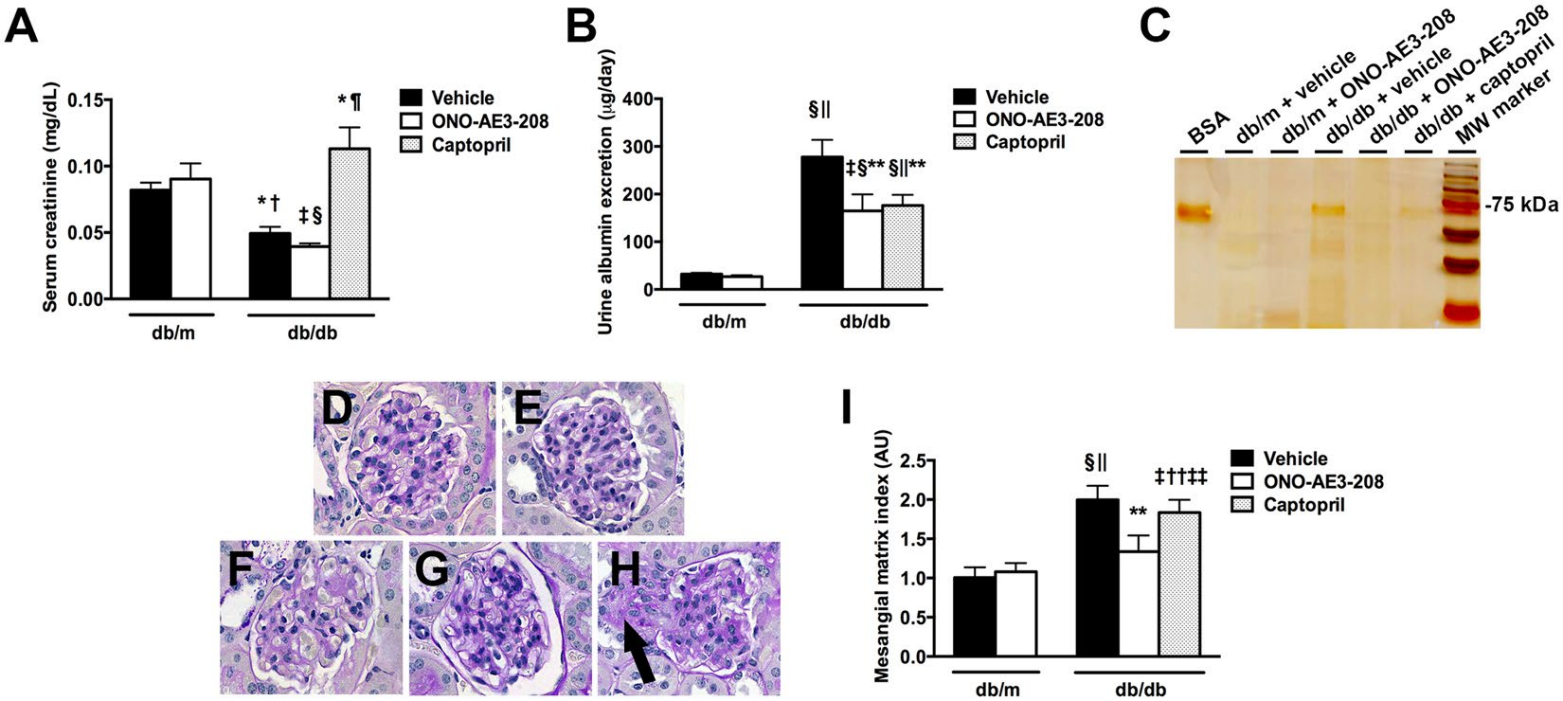
Renal function and mesangial matrix in non-diabetic db/m and diabetic db/db mice treated with vehicle (drinking water) or ONO-AE3-208 for eight weeks or db/db mice treated with captopril for eight weeks. db/m + vehicle n = 12, db/m + ONO-AE3-208 n = 12, db/db + vehicle n = 10, db/db + ONO-AE3-208 n = 7, db/db + captopril n = 8. (**A**) Serum creatinine. (**B**) Urine albumin excretion. (**C**) Silver-stained polyacrylamide gel of urine samples from db/m and db/db mice treated with vehicle, ONO-AE3-208 or captopril, after loading according to urine creatinine concentration. (**D**–**H**) Representative photomicrographs of periodic acid-Schiff stained kidney sections from (**D**) db/m + vehicle, (**E**) db/m + ONO-AE3-208, (**F**) db/db + vehicle, (**G**) db/db + ONO-AE3-208, (**H**) db/db + captopril. Original magnification ×400. The arrow points to a hyperplastic juxtaglomerular apparatus in the kidney section from the db/db mouse treated with captopril. (**I**) Mesangial matrix index. AU = arbitrary units. BSA = bovine serum albumin (1 µg), MW = molecular weight. *p < 0.05 vs. db/m + vehicle; ^†^p < 0.01 vs. db/m + ONO-AE3-208; ^‡^p < 0.001 vs. db/m + vehicle; ^§^p < 0.0001 vs. db/m + ONO-AE3-208; ^¶^p < 0.0001 vs. db/db + vehicle or db/db + ONO-AE3-208; ^||^p < 0.0001 vs. db/m + vehicle; ^**^p < 0.01 vs. db/db + vehicle; ^††^p < 0.001 vs. db/m + ONO-AE3-208; ^‡‡^p < 0.05 vs. db/db + ONO-AE3-208.

Although our experiments performed in STZ-eNOS^−/−^ mice enabled us to detect an anti-albuminuric effect of ONO-AE3-208, we were cognizant of limitations of this experiment including the short duration of study (three weeks) and the confounding effects of eNOS absence. Thus, we decided to perform a second study, this time administering ONO-AE3-208 to Type 2 diabetic db/db mice, one of the most commonly studied mouse models of diabetes. We treated non-diabetic db/m mice and Type 2 diabetic db/db mice with ONO-AE3-208 or vehicle for eight weeks and, in this study, we compared the effects of ONO-AE3-208 with “standard of care” by treating an additional group of db/db mice with the ACE inhibitor, captopril (Table 2). There was no difference in blood glucose between db/db mice treated with vehicle, ONO-AE3-208 and captopril (Table 2). As expected, systolic blood pressure (SBP) was lower in db/db mice treated with captopril than db/db mice treated with vehicle, whereas systemic pressure was not affected by ONO-AE3-208 (Table 2). Serum creatinine was decreased in vehicle-treated db/db mice in comparison to db/m mice, consistent with hyperfiltration (Fig. 3A). Captopril prevented the decrease in creatinine in db/db mice whereas ONO-AE3-208 had no effect (Fig. 3A). Despite this difference in serum creatinine, albuminuria was reduced with both ONO-AE3-208 and captopril (Fig. 3B and C). Histologically, consistent with the marked lowering of blood pressure with ACE inhibition, the kidneys of captopril-treated db/db mice showed pronounced juxtaglomerular hyperplasia, which was not observed in any of the other study groups (Fig. 3D–I). Further highlighting the difference in effect of EP4 inhibition and ACE inhibition, mesangial matrix accumulation was reduced with ONO-AE3-208 but not with captopril (Fig. 3D–I).

**Table 2:** Metabolic characteristics of db/m and db/db mice treated with vehicle (drinking water) or ONO-AE3-208 (10 mg/kg/day) or db/db mice treated with captopril (20 mg/kg/day) for eight weeks.

	*n*	Body weight (g)	Mean kidney weight (g)	Mean kidney weight: body weight (%)	Systolic blood pressure (mmHg)	Blood glucose (mmol/L)
db/m	12	30.7 ± 0.6	0.199 ± 0.003	0.65 ± 0.01	90 ± 5	7.8 ± 0.3
db/m + ONO-AE3-208	12	29.2 ± 0.6	0.204 ± 0.006	0.70 ± 0.03	97 ± 3	8.0 ± 0.4
db/db	10	37.9 ± 1.1^ab^	0.237 ± 0.005^af^	0.63 ± 0.03	104 ± 6^d^	29.7 ± 1.0^ab^
db/db + ONO-AE3-208	7	36.6 ± 1.9^bc^	0.212 ± 0.012^g^	0.57 ± 0.47	98 ± 5	28.8 ± 1.0^ab^
db/db + captopril	8	34.5 ± 2.0^de^	0.233 ± 0.008^fh^	0.64 ± 0.04	80 ± 6^ijk^	28.9 ± 1.3^ab^

^a^p < 0.0001 vs. db/m, ^b^p < 0.0001 vs. db/m + ONO-AE3-208, ^c^p < 0.01 vs. db/m, ^d^p < 0.05 vs. db/m, ^e^p < 0.01 vs. db/m + ONO-AE3-208, ^f^p < 0.001 vs. db/m + ONO-AE3-208, ^g^p < 0.05 vs. db/db, ^h^p < 0.001 vs. db/m, ^i^p < 0.05 vs. db/m + ONO-AE3-208, ^j^p < 0.01 vs. db/db, ^k^p < 0.05 vs. db/db + ONO-AE3-208.

### ONO-AE3-208 attenuates kidney injury in subtotally nephrectomized rats

**Figure 4:**
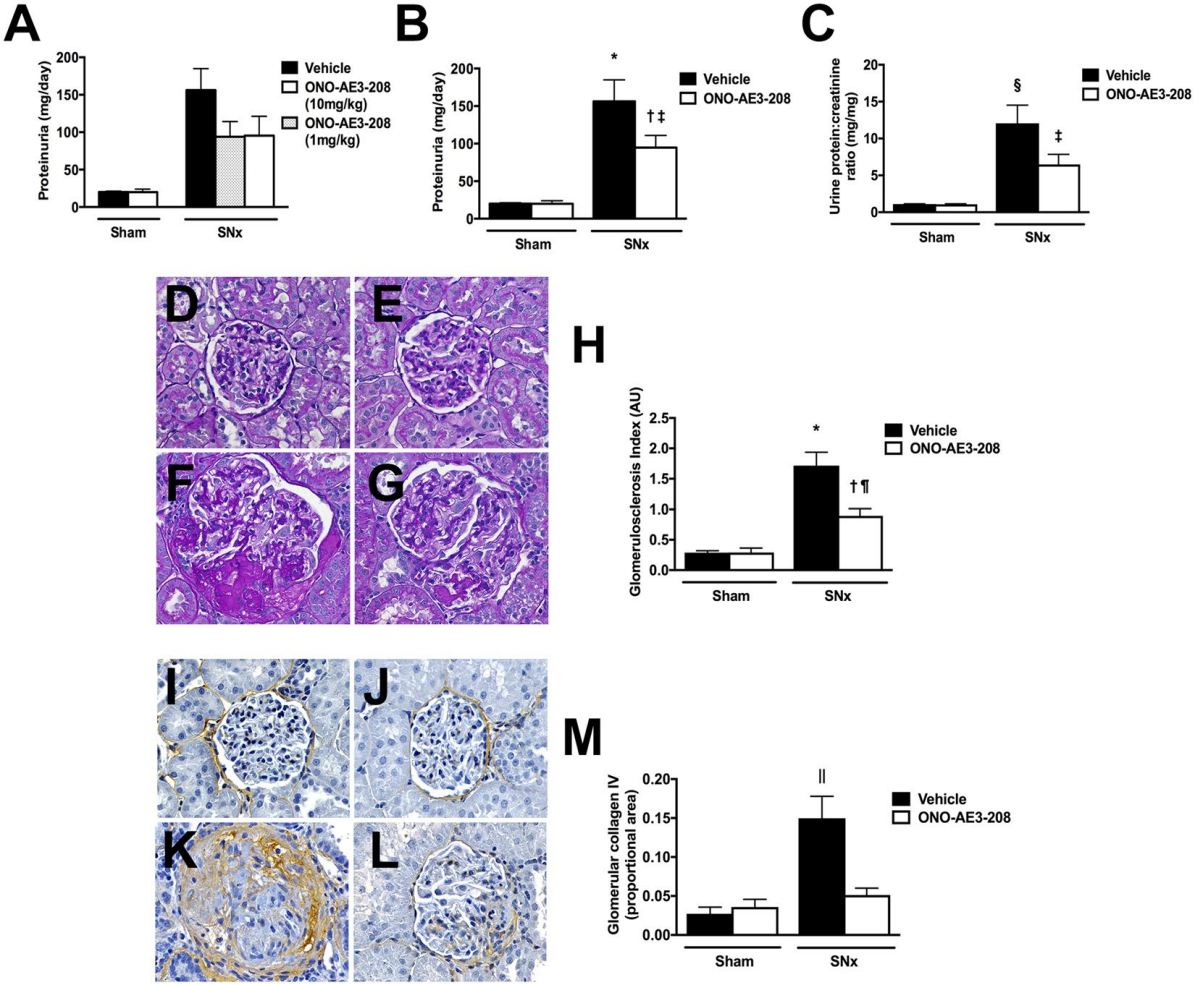
Urine protein excretion and glomerular injury in sham and subtotally nephrectomized (SNx) rats treated with vehicle (drinking water) or ONO-AE3-208 for seven weeks. Sham + vehicle n = 12, sham + ONO-AE3-208 n = 12, SNx + vehicle n = 18, SNx + ONO-AE3-208 (1 mg/kg/day) n = 8, SNx + ONO-AE3-208 (10 mg/kg/day) n = 8. (**A**) Increased 24 hour urine protein excretion in SNx rats and an equivalent reduction with either 1 mg/kg/day or 10 mg/kg/day ONO-AE3-208. Because of this equivalent reduction, the two groups of ONO-AE3-208-treated SNx rats were subsequently combined in further analyses. (**B**) 24 hour urine protein excretion. (C) Urine protein:creatinine ratio. (**D**–**G**) Representative photomicrographs of periodic acid-Schiff stained kidney sections from (**D**) sham + vehicle, (**E**) sham + ONO-AE3-208, (**F**) SNx + vehicle, (**G**) SNx + ONO-AE3-208. Original magnification ×400. (**H**) Glomerulosclerosis index. (**I**–**L**) Representative photomicrographs of collagen IV immunostained kidney sections from (**I**) sham + vehicle, (**J**) sham + ONO-AE3-208, (**K**) SNx + vehicle, (**L**) SNx + ONO-AE3-208. Original magnification ×400. (**M**) Proportional glomerular area positively immunostaining for collagen IV. AU = arbitrary units. *p < 0.0001 vs. sham + vehicle or sham + ONO-AE3-208; ^†^p < 0.05 vs. sham + vehicle or sham + ONO-AE3-208; ^‡^p < 0.05 vs. SNx + vehicle; ^§^p < 0.001 vs. sham + vehicle or sham + ONO-AE3-208; ^¶^p < 0.001 vs. SNx + vehicle, ^||^p < 0.0001 vs. all other groups.

Whereas the db/db mouse model is widely studied as a model of diabetic kidney disease this model is also restricted in its applicability because db/db mice do not develop advanced glomerulosclerosis with glomerular filtration rate (GFR) decline as may be observed in patients. Indeed the renoprotective effects of ACE inhibition, now routinely used in diabetes management, were initially examined in a non-diabetic experimental model of raised intraglomerular pressure: the subtotally nephrectomized (SNx) rat^19^. Accordingly, to determine whether the reno-protective actions of ONO-AE3-208 extend to more advanced stages of nephropathy and to also determine whether the reno-protective effects of ONO-AE3-208 are glucose-dependent, we tested the effects of the EP4 inhibitor in SNx rats. Male Sprague Dawley rats underwent sham or SNx surgery and, one week later, were randomly allocated to receive either drinking water or ONO-AE3-208 in drinking water for seven weeks. In this series of experiments, we elected to compare the effects of ONO-AE3-208 when administered at two different doses: 1 mg/kg/day and 10 mg/kg/day. SBP was increased and GFR was reduced in SNx rats in comparison to sham-operated animals and neither dose of ONO-AE3-208 had an effect on either of these parameters (Table 3). In contrast, proteinuria (which was increased in SNx rats) was equivalently reduced with both 1 mg/kg/day and 10 mg/kg/day of ONO-AE3-208 (Fig. 4A–C). Because the anti-proteinuric effects were equivalent for the two doses of ONO-AE3-208, we combined the two treatment groups for histological analysis. Paralleling the changes in proteinuria, the magnitude of glomerular scarring, when assessed either on periodic acid-Schiff stained kidney sections (Fig. 4D–H) or following immunostaining for collagen IV (Fig. 4I–M), was increased in vehicle-treated SNx rats and was attenuated with ONO-AE3-208.

**Table 3:** Metabolic characteristics of sham-operated and subtotally nephrectomized (SNx) rats treated with vehicle (drinking water) or ONO-AE3-208 for seven weeks.

	*n*	Body weight (g)	Left kidney weight (g)	Left kidney weight:body weight (%)	Systolic blood pressure (mmHg)	GFR (ml/min/kg)
Sham	12	617 ± 15	1.678 ± 0.037	0.28 ± 0.01	125 ± 2	7.8 ± 0.5
Sham + ONO-AE3-208 (10 mg/kg/day)	12	593 ± 17	1.732 ± 0.058	0.29 ± 0.01	132 ± 3	8.2 ± 0.7
SNx	18	516 ± 15^ab^	2.319 ± 0.068^af^	0.45 ± 0.01^af^	196 ± 11^af^	2.4 ± 0.4^af^
SNx + ONO-AE3-208 (1 mg/kg/day)	8	510 ± 20^bc^	2.084 ± 0.129^bdg^	0.41 ± 0.02^ah^	184 ± 9^ah^	3.1 ± 0.7^af^
SNx + ONO-AE3-208 (10 mg/kg/day)	8	525 ± 23^de^	2.052 ± 0.108^bdg^	0.36 ± 0.04^dei^	181 ± 5^ah^	3.2 ± 0.6^af^

^a^p < 0.0001 vs. sham, ^b^p < 0.01 vs. sham + ONO-AE3-208, ^c^p < 0.001 vs. sham, ^d^p < 0.01 vs. sham, ^e^p < 0.05 vs. sham + ONO-AE3-208, ^f^p < 0.0001 vs. sham + ONO-AE3-208, ^g^p < 0.05 vs. SNx, ^h^p < 0.001 vs. sham + ONO-AE3-208, ^i^p < 0.001 vs. SNx.

### ONO-AE3-208 prevents podocyte dedifferentiation induced by transforming growth factor-ß1 (TGF-ß1)

**Figure 5:**
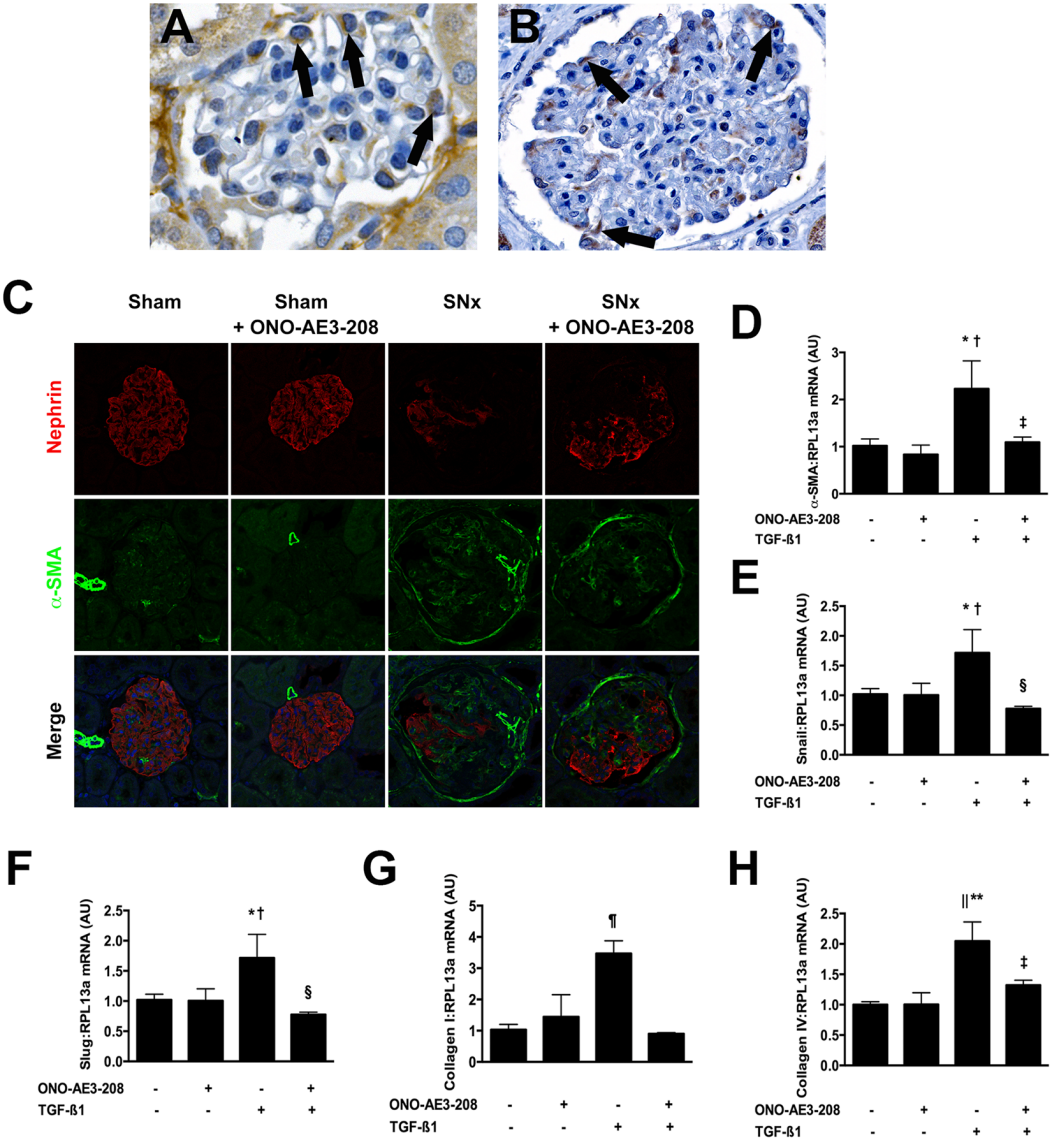
Presence and actions of EP4 in glomerular podocytes. (**A**,**B**) Immunostaining demonstrating EP4 protein in peripherally arranged glomerular podocytes from (**A**) a C57BL/6 mouse and (**B**) normal human kidney tissue. Original magnification ×400. The arrows mark positively immunostaining peripherally arranged podocytes. (**C**) Dual immunofluorescence for nephrin (red) and α-smooth muscle actin (α-SMA; green) in glomeruli from sham-operated and subtotally nephrectomized (SNx) rats treated with vehicle or ONO-AE3-208. Blue = DAPI. (**D**–**H**) Effect of ONO-AE3-208 on TGF-ß1-induced dedifferentiation of cultured mouse podocytes. (**D**) α-SMA mRNA levels. (**E**) Snail mRNA levels. (**F**) Slug mRNA levels. (**G**) Collagen I mRNA levels. (**H**) Collagen IV mRNA levels. AU = arbitrary units. *p < 0.05 vs. control; ^†^p < 0.05 vs. ONO-AE3-208; ^‡^p < 0.05 vs. TGF-ß1; ^§^p < 0.01 vs. TGF-ß1; ^¶^p < 0.01 vs. all other conditions; ^||^p < 0.01 vs. control; **p < 0.001 vs. ONO-AE3-208.

The difference in the renal effects of ONO-AE3-208 and captopril, despite both being accompanied by an equivalent reduction in albuminuria, suggested to us that the reno-protective effects of EP4 inhibition are not limited to actions on hemodynamic forces. Likewise, the observation that the reno-protective effect of ONO-AE3-208 was also apparent in non-diabetic SNx rats suggested to us that this effect was also not restricted to high glucose mediated events. We thus reasoned that the actions of ONO-AE3-208 may at least in part be mediated by preventing the deleterious effects of dysregulated growth factors or cytokines that are common to both diabetic and non-diabetic CKD and that these effects may occur in resident glomerular cells. We immunostained kidney sections from both mice and humans and we observed prominent expression of EP4 in glomerular podocytes of each species (Fig. 5A,B). By dual immunofluorescence, we observed an accumulation of the mesenchymal marker, α-smooth muscle actin (α-SMA) in the glomeruli of SNx rats, coinciding with segmental loss of the podocyte slit-pore protein, nephrin (Fig. 5C). Collectively, these findings led us to speculate that signaling through the EP4 receptor in podocytes may facilitate their dedifferentiation into more mesenchymal-like cells. Accordingly, in our final experiments we exposed differentiated immortalized mouse podocytes to the pro-fibrotic growth factor TGF-ß1, which is upregulated in both diabetic^20^ and non-diabetic kidney disease^21^. TGF-ß1 caused the dedifferentiation of cultured podocytes as reflected by an upregulation in the expression of α-SMA, snail, slug, collagen I and collagen IV whereas this effect was negated by pre-treatment of podocytes with ONO-AE3-208 (Fig. 5D–H).

## Discussion

Despite recent advances^22^, new treatments are still urgently needed for people with CKD. For example, for people with diabetes, a diagnosis of CKD more than doubles healthcare costs^23, 24^ and even for individuals with Stage 3A kidney disease (GFR 45-59 ml/min) all-cause mortality is increased four-fold^25^. Here, in an effort to examine new treatment opportunities for CKD we explored the effect of pharmacological antagonism of the PGE_2_, EP4 receptor. Across three different experimental rodent models of diabetic and non-diabetic kidney disease we observed that EP4 inhibition attenuated albuminuria or proteinuria, independent risk markers of renal decline^26^ and mortality^27^. The effects of EP4 antagonism were phenotypically distinct from those of either broadspectrum COX inhibition or ACE inhibition and were not limited to the diabetic state, attenuating glomerular scarring in both Type 2 diabetic db/db mice and non-diabetic rats with progressive proteinuric kidney disease induced by surgical renal mass ablation. In cultured podocytes, EP4 inhibition prevented podocyte dedifferentiation.

EP4 is a typical seven transmembrane domain G-protein coupled receptor, which as a superfamily make up over 50% of all current drug targets^28^. Like the other EP subfamily member, EP2, EP4 is a G_s_α coupled receptor which activates adenylate cyclase to increase cyclic adenylyl monophosphate (cAMP) production on ligand-binding^8, 9^. Its ligand, PGE_2_, is generated via three different PGE_2_ synthases: microsomal prostaglandin E synthase (mPGES)-1, mPGES-2 and cytosolic PGES (cPGES). In rat mesangial cells, mPGES-1 was induced by TGF-ß1^29^ and, more recently, mPGES-1-derived PGE_2_ was shown to contribute to podocyte injury caused by adriamycin^30^. Although in the latter study, the EP receptor subtype responsible for promoting podocyte injury in response to adriamycin was not established, an earlier study employing podocyte-specific EP4 overexpressing or deficient mice pointed to a deleterious function of the EP4 subtype receptor in podocytes of mice subjected to renal mass ablation^13^. On the background of this collective body of evidence, we sought to examine the effects of EP4 inhibition on the development of glomerular disease in CKD.

We elected to first examine the effects of EP4 inhibition in STZ-eNOS^−/−^ mice. We selected this mouse model for two reasons. Firstly, we had earlier shown that glomerular injury in STZ-eNOS^−/−^ mice predominantly occurs within podocytes^18^ (which our histological survey revealed are the major glomerular cell-type that expresses EP4). Secondly, the magnitude and pace of albuminuria development in STZ-eNOS^−/−^ mice occurring within two to three weeks of diabetes induction^18, 31^, allows the relatively rapid *in vivo* screening of compounds for possible anti-albuminuric actions^31^. Whereas the EP4 inhibitor ONO-AE3-208 caused an approximate 50% reduction in urinary albumin excretion in STZ-eNOS^−/−^ mice, the broadspectrum COX inhibitor indomethacin made no difference. This latter observation underscores the distinct effects of selective prostanoid receptor subtype targeting. It is possible, for example, that with COX inhibition, concurrent blockade of the synthesis of other prostanoids (e.g. prostaglandin I_2_^31^) or concurrent prevention of the activation of other prostanoid receptors (e.g. EP2^32^) negates the apparently beneficial effects observed with selective PGE_2_-EP4 targeting.

The anti-albuminuric effect that we observed in STZ-eNOS^−/−^ mice was subsequently replicated in Type 2 diabetic db/db mice that were treated with ONO-AE3-208 for eight weeks and, in this longer duration study, was accompanied by attenuated mesangial matrix accumulation. One of the principal mechanisms by which glomerular injury occurs in diabetes is through glomerular hypertension^33^. Indeed, the reno-protective effects of ACE inhibitors are considered to be, at least in part, mediated by a diminution of intraglomerular pressure caused by preferential dilatation of the efferent arteriole^34^. In contrast, EP4 appears to play an important role in sustaining vasodilatation of the preglomerular afferent arteriole^35^. In the present study, serum creatinine was lower in db/db mice than non-diabetic db/m mice suggestive of renal hyperfiltration (although GFR was not directly determined in these animals). This reduction in creatinine was normalized by the ACE inhibitor captopril suggestive of a reduction in intraglomerular pressure. However, it was unaltered by ONO-AE3-208. Thus, the effects of ONO-AE3-208 on albuminuria and matrix deposition in db/db mice are unlikely to be a consequence of renal hemodynamic actions. GFR was similarly unaffected by EP4 inhibition in SNx rats, where it was reduced in comparison to control animals and in contrast to the diabetic mouse study. Despite this lack of effect on GFR with EP4 inhibition, proteinuria and glomerular injury were both significantly reduced with ONO-AE3-208 in SNx rats. This disparity is not surprising and is reminiscent of the actions of ACE inhibition in this model. For instance, in the original report published over 30 years ago, treatment of subtotally nephrectomized Munich-Wistar rats with the ACE inhibitor enalapril reduced proteinuria and glomerular scarring without affecting single nephron GFR^19^.

As stated at the outset, in the opinion of the investigators it is unlikely that the reno-protective actions of EP4 inhibition that were observed during the present series of experiments can be attributed to effects on a single pathway in a single renal cell-type. However, the collective observations suggest that the effects may be, at least partly, mediated by direct actions on resident glomerular cells. Consistent with this supposition, in cultured podocytes EP4 inhibition prevented TGF-ß1-induced podocyte dedifferentiation. Whereas EP4 is recognized as a G_s_α-coupled receptor, the intracellular signaling pathways that it initiates are more complex than this. For example, EP4 is also coupled with G_i_α^36^ and this relationship may explain the comparatively lower ability of EP4 signaling to raise cAMP levels compared to EP2^9^. Through increasing cAMP levels, EP4 can activate both protein kinase A (PKA) and exchange protein directly activated by cAMP (Epac)^9^. Activation of the receptor can also induce intracellular signaling through a number of cAMP-independent second messengers, including phosphatidylinositol 3-kinase (PI3K)^9^, extracellular signal-regulated kinase (ERK)^9^, and p38 mitogen-activated protein kinase (p38 MAPK)^37^. Thus, there are several potential, and by no means mutually exclusive, points of convergence for TGF-ß1- and EP4-regulated pathways. In prostate cancer cells, for example, EP4 inhibition prevented TGF-ß signaling through the PI3K/Akt pathway^38^, whereas PI3K/Akt^39, 40^, ERK^40^ and p38 MAPK^41^ have each been implicated in TGF-ß1-induced epithelial mesenchymal transition.

The present report has weaknesses. Firstly, as emphasized above, the precise means by which EP4 inhibition prevented kidney or podocyte injury in the experiments herein described remains uncertain. Secondly, whilst the findings are consistent with some reports (e.g. refs 11, 13 and 15) they are at variance with others^14^. However, a strength of the study is the evaluation of the actions of one EP4 inhibitor in multiple different models of kidney disease. In the mouse and rat models of diabetic and non-diabetic kidney disease studied and with the particular small molecule (ONO-AE3-208) administered for the dose and duration described, EP4 inhibition consistently resulted in a preservation of glomerular permselectivity and a prevention of renal injury. Existing therapies for CKD, e.g. RAS-blockade^42^ and sodium-glucose cotransporter 2 inhibition^22^, likely confer renoprotection through actions at both the cell and organ level and it seems reasonable to presume that the effects of EP4 inhibition are similarly broad. Amongst these broad effects, is the preservation of podocyte differentiation when challenged by the pro-fibrotic cytokine, TGF-ß1.

In summary, in rodent models of diabetic and non-diabetic CKD, EP4 inhibition attenuated renal injury through mechanisms that were distinct from either broadspectrum COX inhibition or RAS-blockade. EP4 inhibitors have reached clinical trial for other indications^43^ and may offer a viable repurposing opportunity for the treatment of CKD.

## Methods

### Animal studies

#### Streptozotocin (STZ)-diabetic eNOS^−/−^ mice

Male C57BL/6 and eNOS^−/−^ (C57BL/6 genetic background) mice (The Jackson Laboratory, Bar Harbor, ME) were studied at eight weeks of age. Mice received a daily i.p. injection of STZ (55 mg/kg in 0.1 M citrate buffer, pH 4.5) or citrate buffer alone after a 4 hour fast for five consecutive days. Animals received ONO-AE3-208 (Medchemexpress, Monmouth Junction, NJ) at a dose of 10 mg/kg/day in drinking water^17^ or drinking water alone, for three weeks beginning on the day of the first injection of STZ. In a previous report, ONO-AE3-208 administered orally to mice as a 10 mg/kg bolus achieved a peak plasma concentration of 677 ng/ml after 0.25 hours with 18% bioavailability^17^. Urine nephrin content (Exocell, Philadelphia, PA) and urine albumin excretion (Assaypro, St. Charles, MO) were determined by ELISA after housing mice in individual metabolic cages for 24 hours. Blood glucose was determined by OneTouch UltraMini (LifeScan Canada Ltd., Burnaby, British Columbia, Canada). To determine the effect of broadspectrum COX inhibition, male control and STZ-diabetic/6 and eNOS^−/−^ mice were treated with either indomethacin (4 mg/kg/day in drinking water^44^, Cayman Chemical, Ann Arbor, MI) or drinking water alone beginning with the first i.p. injection of STZ and continued for two weeks (n = 10/group).

#### db/db mice

Male db/m and db/db mice on a BKS background (The Jackson Laboratory) aged eight weeks were randomly allocated to receive either ONO-AE3-208 (10 mg/kg/day in drinking water) or drinking water alone for eight weeks. An additional group of db/db mice were treated contemporaneously with captopril (Sigma-Aldrich, Oakville, Ontario, Canada) at a dose of 20 mg/kg/day in drinking water^18^. Blood glucose and urine albumin excretion were determined as already described. SBP was determined using a CODA non-invasive blood pressure system (Kent Scientific, Torrington, CT)^18^. Serum creatinine was determined by HPLC (Vanderbilt University, Nashville, TN). For silver staining, urine volumes containing 0.5 µg creatinine (Creatinine Companion, Exocell, Philadelphia, PA) were solubilized in sample buffer (ThermoFisher Scientific, Rockford, IL) and separated by SDS-PAGE before staining with a ProteoSilver Stain kit (Sigma Aldrich).

#### Subtotally nephrectomized rats

Male Sprague Dawley rats (Charles River, Montreal, Quebec) aged eight weeks underwent sham or subtotal nephrectomy surgery as previously described^45^. Briefly, for subtotal nephrectomy surgeries, under isoflurane anesthesia, the right kidney was removed via subcapsular nephrectomy and infarction of two thirds of the left kidney was achieved by selective ligation of two out of three of the branches of the left renal artery. Sham surgery involved laparotomy and manipulation of both kidneys prior to wound closure. One week later, rats were randomized to receive ONO-AE3-208 (1 mg/kg/day or 10 mg/kg/day) in drinking water or drinking water alone and they were followed for a further seven weeks. SBP was determined by tail cuff plethysmography (Powerlab, ADInstruments, Colorado Springs, CO) as previously described^46^. GFR was determined by single shot FITC inulin clearance with repeated sampling via the tail vein as previously described^45^. Urine protein excretion was determined using the benzethonium chloride method after 24 hour metabolic caging and urine creatinine was determined by autoanalyzer (Advia 1650, Siemens Medical Solutions Diagnostics, Tarrytown, NY).

Mouse and rat kidney tissue was immersion-fixed in 10% neutral buffered formalin before routine processing and sectioning. All experimental procedures adhered to the guidelines of the Canadian Council on Animal Care and were approved by the St. Michael’s Hospital Animal Care Committee, Toronto, Ontario, Canada.

### Glomerular volume

Glomerular volume (Gv) was calculated on 4 μm periodic acid Schiff-stained kidney sections using the formula:

GV = (ß/k)(GA)^3/2^

where ß = 1.38 pertains to the sphere and k = 1.10 is the distribution coefficient^47^.

### Mesangial matrix index and glomerulosclerosis index

The magnitude of mesangial matrix deposition in the diabetic mouse studies or glomerulosclerosis in the SNx rat study was determined on periodic acid-Schiff stained kidney sections using a semi-quantitative scoring method on approximately 50 glomerular profiles per kidney section as previously described^31, 45^.

### Human studies

Formalin-fixed paraffin-embedded kidney tissue was examined from three cadaveric donors with no prior history of kidney disease (National Disease Research Interchange (NDRI), Philadelphia, PA). The study was approved by the Research Ethics Board of St. Michael’s Hospital.

### Immunohistochemistry

Immunohistochemistry was performed with antibodies in the following concentrations: collagen IV 1:500 (EMD Millipore, Darmstadt, Germany); EP4 1:100 (Cayman Chemical). Incubation with phosphate buffered saline in place of the primary antibody served as the negative control. After incubation with the appropriate horseradish peroxidase conjugated secondary antibody, sections were labeled with Liquid Diaminobenzidine and Substrate Chromogen (Dako North America Inc., Carpinteria, CA) before counterstaining in Mayer’s hematoxylin. For quantitation of glomerular collagen IV in rat kidney sections, slides were scanned with the Aperio ScanScope System (Aperio Technologies Inc., Vista, CA). The proportional glomerular area positively immunostaining for collagen IV was determined in 30 randomly selected glomerular profiles from each kidney section using ImageScope (Aperio Technologies Inc.).

### Dual immunofluorescence

Immunofluorescence microscopy was performed on paraffin embedded kidney sections with antibodies in the following concentrations: α-SMA 1:200 (Abcam, Cambridge, MA), secondary antibody Alexa Fluor 488 donkey anti-rabbit IgG 1:200 (ThermoFisher Scientific) and nephrin 1:200 (R & D Systems, Minneapolis, MN), secondary antibody Alexa Fluor 647 donkey anti-goat 1:200 (Abcam). DAPI was from Cell Signaling Technology (Danvers, MA) and was used at a concentration of 1:10,000. Slides were visualized on a Zeiss LSM 700 confocal microscope (Carl Zeiss Canada, Toronto, Ontario, Canada).

### Cultured mouse podocytes

Differentiated immortalized mouse podocytes were cultured as previously described^48^. Cells were serum starved for 16 hours before incubation with ONO-AE3-208 (1 µM^49^) or vehicle (DMSO) for 1 hour before supplementation of the medium with 10 ng/ml recombinant TGF-ß1 (BioRad Laboratories Inc., Hercules, CA) for 48 hours. In a previous study of EP4 overexpressing human colonic epithelial cells, incubation with 1 µM ONO-AE3-208 resulted in a >90% reduction in PGE_2_-induced luciferase activity, whereas the response in EP2 overexpressing cells was unaffected^49^. RNA was isolated from cell extracts using TRIzol Reagent (ThermoFisher Scientific). SuperScript III Reverse Transcriptase (ThermoFisher Scientific) was used to reverse transcribe cDNA from 1 µg RNA. Primers were designed and validated using Primer-BLAST (http://www.ncbi.nlm.nih.gov/tools/primer-blast/) and were synthesized by Integrated DNA Technologies (Coralville, IA). Primer sequences were as follows: α-SMA forward CAGGGAGTAATGGTTGGAAT, reverse TCTCAAACATAATCTGGGTCA; snail forward GCCGGAAGCCCAACTATAGCGA, reverse TTCAGAGCGCCCAGGCTGAGGTACT; slug forward TGTTGCAGTGAGGGCAAGAA, reverse GACCCTGGTTGCTTCAAGGA; collagen I forward CAGTCGATTCACCTACAGCACG, reverse GGGATGGAGGGAGTTTACACG; collagen IV forward AGGACTTCCCGGCTTGAATG, reverse TCCCCCTTATCTCCTTGGGG; RPL13a forward GCTCTCAAGGTTGTTCGGCTGA, reverse AGATCTGCTTCTTCTTCCGATA. Gene expression was determined using SYBR green (Wisent Inc, St. Bruno, Quebec, Canada) on a ViiA7 Real-Time PCR System (ThermoFisher Scientific). Experiments were performed in triplicate and data analyses were performed using the Applied Biosystems Comparative C_T_ method.

### Statistical analysis

Data are expressed as means ±  SEMs. Statistical significance was determined by one-way ANOVA with a Fisher’s least significant difference test. Statistical analyses were performed using GraphPad Prism 6 for Mac OS X (GraphPad Software Inc., San Diego, CA).
